# Treatment Adherence and Health-Related Quality of Life in Patients with Hemophilia in Hong Kong

**DOI:** 10.3390/ijerph19116496

**Published:** 2022-05-26

**Authors:** Yin Ting Cheung, Pok Hong Lam, Henry Hon Wai Lam, Chung-Tin Ma, Alex Wing Kwan Leung, Raymond Siu Ming Wong, Chi Kong Li

**Affiliations:** 1 School of Pharmacy, Faculty of Medicine, The Chinese University of Hong Kong, Hong Kong, China; justinma@link.cuhk.edu.hk; 2 Department of Paediatrics, Faculty of Medicine, The Chinese University of Hong Kong, Hong Kong, China; calvinlam@link.cuhk.edu.hk (P.H.L.); alexwkleung@cuhk.edu.hk (A.W.K.L.); ckli@cuhk.edu.hk (C.K.L.); 3 Hong Kong Haemophilia Society, Hong Kong, China; henry.lam@haemophilia.org.hk; 4 Department of Paediatrics & Adolescent Medicine, Hong Kong Children’s Hospital, Hong Kong, China; 5 Hong Kong Hub of Paediatric Excellence, The Chinese University of Hong Kong, Hong Kong, China; 6 Department of Medicine and Therapeutics, Faculty of Medicine, The Chinese University of Hong Kong, Hong Kong, China; raymondwong@cuhk.edu.hk

**Keywords:** hemophilia, quality of life, treatment adherence, supportive care, chronic illness

## Abstract

Background: This study aims to identify factors affecting health-related quality of life (HRQoL) in Chinese patients with hemophilia in Hong Kong, and to examine the association between treatment adherence and HRQoL outcomes. Methods: Patients with hemophilia A or B from a non-governmental organization reported their HRQoL and treatment adherence to prophylactic therapy using validated tools. Univariate tests and multivariable regression analysis were used to compare differences in outcomes across clinically relevant subgroups. Results: Fifty-six patients were recruited (mean age 30.4 [17.4] years; majority hemophilia A: 75%; moderate-to-severe severity: 88%). Patients who received prophylactic treatment reported fewer work/school problems (25.8 [18.9] versus 51.5 [26.3]; *p* = 0.001) than those who received on-demand therapy. The multivariable model showed that older age (*B* = 0.42, 95% CI = 0.093–0.75) and living in public housing (*B* = 10.24, 95% CI = 0.70–19.77) were associated with worse HRQoL. Older age was associated with treatment non-adherence (*r* = 0.66, *p* < 0.0001). Patients with poor adherence tended to report worse functioning in sports/leisure (*r* = 0.31, *p* = 0.033). Conclusions: Our results suggest that patients who were older, had lower education attainment and received on-demand treatment had poorer perception of their health. Improving adherence may lead to better HRQoL. Future work includes evaluating the occupational needs prospectively in this population.

## 1. Introduction

Hemophilia is a group of inherited hemorrhagic disorders primarily affecting males [1]. Hemophilia A is caused by the deficiency of coagulation factor VIII, while Hemophilia B is characterized by the deficiency of coagulation factor IX [1], Hemophilia C refers to factor XI deficiency, which is caused by mutations to the F11 gene and can occur in males and females. Hemophilia usually manifests during infancy as spontaneous or post-traumatic bleeding in any part of the body, especially in joints, soft tissue and muscles [2]. The median age at diagnosis is 1 month or younger for individuals with severe hemophilia, approximately 8 months for individuals with moderate hemophilia, and 3 years for individuals with mild hemophilia [3]. The suboptimal treatment of hemophilia may lead to long-term irreversible joint deformities and muscle atrophy, resulting in limitations to daily activities and physical functioning [4,5,6]. As most patients with hemophilia require lifelong management of their conditions and complications, studies showed that they tend to have impaired functional status, frequent hospitalization or absenteeism from school or work, and a compromised health-related quality of life (HRQoL) [2,5,6,7,8,9,10,11].

Fortunately, advances in treatment modalities have reduced the mortality and complications associated with hemophilia [2,12]. In general, on-demand treatment with clotting factors is given in the cases of acute bleeding or trauma, while prophylactic clotting factor replacement therapy has been found to be effective in preventing bleeding and reducing long-term complications, such as chronic arthropathy [2,4,12,13]. Current guidelines recommend that whenever appropriate and possible, patients with moderate-to-severe hemophilia should self-administer prophylactic clotting factors at home, either by themselves or with the help of their caregivers, and have regular follow-ups at a hemophilia clinic [14]. Despite the advantages of prophylactic clotting factors, studies have reported a low adherence to home-based therapy in patients with hemophilia, due to significant barriers and perceived limitations [9,15,16], including infusion pain, difficulty with venous access and the time-consuming nature of the procedure [9,15,16]. Better treatment adherence was a significant predictor of less bodily pain and better social functioning in patients with hemophilia [17,18]. These findings collectively suggest that more attention should be paid to identifying patients with hemophilia who are non-adherent to medication.

In Hong Kong, approximately 222 mild-to-severe hemophilia patients (192 Hemophilia A and 30 Hemophilia B) under regular public care in Hong Kong under the care of the public healthcare system [19]. The distribution of disease severity (43% severe, 33% moderate, and 24% mild) is similar to that of mainland China and Taiwan. Notably, the rate of prophylactic factor infusion (34%) among patient with hemophilia in Hong Kong is slightly higher than the estimated rate (16.2%) in a report from mainland China [20]. The population factor use of 1.83 units of FVIII per capita [19] is comparable to factor consumption levels in other developed countries in Asia, such as Korea, Japan, and Singapore [21]. Following the Government of the Hong Kong Special Administrative Region’s stated commitment in its 2017 Policy Address to focus on healthcare support and acquisition of life-altering drugs for patients with rare diseases [22], much effort is now directed toward providing resources to improve these patients’ HRQoL and functional independence.

To date, no study has systematically identified factors affecting treatment adherence and functional outcomes in Chinese patients with hemophilia in Hong Kong. Therefore, the primary objective of this study was to identify factors affecting HRQoL in patients with hemophilia in Hong Kong, and the secondary objective was to examine the association between treatment adherence and HRQoL outcomes in this population. The findings of this study are expected to guide the development of patient-centered rehabilitation interventions for improving hemophilia patients’ self-efficacy in health management.

## 2. Methods

Between October 2019 and April 2020, participants were recruited using consecutive sampling from the Hong Kong Hemophilia Society, the only active non-governmental organization (NGO) in Hong Kong that provides services for patients with hemophilia. The patients were invited to participate in the study during two NGO events. Eligible participants included patients diagnosed with hemophilia A or B by an adult or pediatric hematologist and who were able to read Chinese or English. Members of the NGO who identified themselves as being diagnosed with non-hemophilia disorders (e.g., von Willebrand disorder) were excluded. The parent(s) of pediatric patients (<18 years of age) participated in the study on their behalf. Written consent was obtained from all participants. The Chinese University of Hong Kong Survey and Behavioral Research Ethics Committee approved this study before its inception (Ref: SBRE-18-052). This study was conducted in accordance with the Declaration of Helsinki.

### 2.1. Study Procedures

On recruitment, the participants completed a structured questionnaire that collected their demographic and medical information, such as age, diagnosis, type of treatment and disease severity. Socioeconomic information, including housing type and highest educational attainment of adult patients (≥18 years) and of the parents of pediatric patients (<18 years) was also collected.

### 2.2. HRQoL Assessment Tool

The HRQoL of adult patients was evaluated using the Haemophilia Quality of Life Questionnaire for Adults (Haem-A-QoL) [23,24], and that of pediatric patients was evaluated using the Hemophilia Quality of Life Questionnaire for Children Short Form (Haemo-QoL-SF), which they completed with their parents’ assistance [25,26]. The English versions of the Haemo-QoL-SF were validated in children with satisfactory internal consistency (Cronbach’s α 0.60 to 0.79) [25,27]. The Haem-A-QoL also demonstrated acceptable psychometric properties (Cronbach’s α 0.64 to 0.95) in adults with hemophilia [23,24,28]. The HRQoL domains measured in these questionnaires are “Physical Health,” “Feelings,” “View of Yourself,” “Sports and Leisure,” “Work and School,” “Dealing with Hemophilia,” “Treatment,” “Future,” and “Family”. Each item is scored from 1 = “never (0% of the time)” to 5 = “always (100% of the time)”. Semantically negative items are reverse-scored. A higher domain or total score is indicative of a worse HRQoL in that domain or overall, respectively. To our knowledge, no study has validated these questionnaires in pediatric and adult patients with hemophilia in Hong Kong. However, one Chinese study validated the Haem-A-QoL in patients with hemophilia in mainland China; this demonstrated that it could differentiate between patients with different hemophilia severity, bleeding status and comorbidities [29].

### 2.3. Adherence Measurement Tool

Among patients who received prophylactic treatment, adherence to treatment was evaluated using the Validated Hemophilia Regimen Treatment Adherence Scale–Prophylaxis (VERITAS-Pro) [30]. This is a 24-item questionnaire comprising six subscales: “Time,” “Dose,” “Plan,” “Remember,” “Skip”, and “Communicate”. Each item is scored from 1 = “always (100% of the time)” to 5 = “never (0% of the time)”. A higher total score is indicative of poorer adherence to treatment. A total score higher than 57 points is considered to indicate significant non-adherence [30]. The VERITAS-Pro was found to have good internal consistency (Cronbach’s α 0.70 to 0.94) and test-retest reliability (Pearson *r* 0.64 to 0.77), and correlated well with infusion logs/diaries [30]. The developers of this instrument provided approval for the use of the simplified Chinese version of the tool. The investigators translated it into traditional Chinese and adapted some terminology to the local population. For example, the term “hemophilia centers” was replaced with “hemophilia clinics” as the healthcare system in Hong Kong does not have the former type of facility.

### 2.4. Statistical Analysis

Study data were de-identified and analyzed using the Statistical Package for the Social Sciences^®^ version 26 (IBM Corp, IBM SPSS Statistics for Macintosh, Armonk, NY, USA). Descriptive statistics were used to summarize the study outcomes (HRQoL and adherence) and baseline characteristics. Categorical variables are presented as absolute frequencies and percentages whereas continuous variables are presented as means and standard deviations (SDs). The analyses of adherence were only conducted in patients who received prophylactic treatment.

Univariate analyses were performed using the Mann–Whitney *U* test (for categorical variables) and Spearman’s correlation test (for continuous variables) to compare differences in study outcomes between patients in different subgroups. The variables included in this study were clinical and socioeconomic factors that have been were hypothesized in the literature to affect HRQoL or adherence: (1) The clinical factors included age as a continuous variable, as well as disease severity (mild-to-moderate versus severe) and treatment type (prophylactic treatment versus on-demand therapy) as categorical variables [6,7,17,24,31]. (2) The socioeconomic factors included housing type (public housing versus private housing) and the highest educational attainment of adult patients and of the parents of pediatric patients (secondary school and below versus post-secondary) as categorical variables [17,18,31,32].

We also conducted two exploratory analyses, as we expected the sample size to be too small to generate conclusive results. First, multivariable linear regression was performed to identify adherence, and clinical and socioeconomic factors that are associated with HRQoL outcomes. Separate models were run for the overall cohort and for adult patients, so that the highest educational attainment of patients could be accounted for by the models. Second, as no study has yet validated the Haem-A-QoL, Haemo-QoL-SF or VERITAS-Pro in patients with hemophilia in Hong Kong, another exploratory analysis was conducted to evaluate the internal consistency and corrected item-total correlation of the subscales using Cronbach’s α and Pearson’s correlation coefficient, respectively [33]. Values greater than 0.7 were considered to be good, between 0.6 and 0.7 as acceptable, and less than 0.6 as weak. For all analyses, a probability (*p*) value of less than 0.05 was regarded as statistically significant.

## 3. Results

Sixty eligible members of the NGO were approached, of whom 58 agreed to participate in the study. Two participants did not complete the questionnaire. Finally, data from 56 patients were analyzed (Response rate: 9.3%; Table 1). The mean age of adult patients (*n* = 42; 75%) was 37.2 (SD = 14.5) years, and that of pediatric patients (*n* = 14; 25%) was 10.0 (SD = 2.8) years. Most patients had hemophilia A (*n* = 42; 75%) with moderate-to-severe disease condition (*n* = 49; 88%). More than one-third of the patients (*n* = 38, 68%) received prophylactic treatment, while the remaining patients (*n* = 17; 30%) received on-demand therapy only, for breakthrough bleeding. Approximately half of the participants lived in public housing (*n* = 26; 46%). The average age of parents of pediatric patients was 41.5 (SD = 6.6) years, and most had attained lower than secondary school education (*n* = 10, 71%).

### 3.1. HRQoL

In the overall cohort, the highest impairment scores (on a scale of 0–100) were reported for the “Sports and Leisure” (mean [SD], 60.5 [24.6]) and “Viewing of Oneself” (48.2 [23.7]) subscales (Supplement Table S1). Relatively lower impairment scores were reported for the “Dealing with Hemophilia” (29.2 [19.1]) and “Work or School” (33.3 [24.4]) subscales (Supplement Table S1).

**Figure 1:**
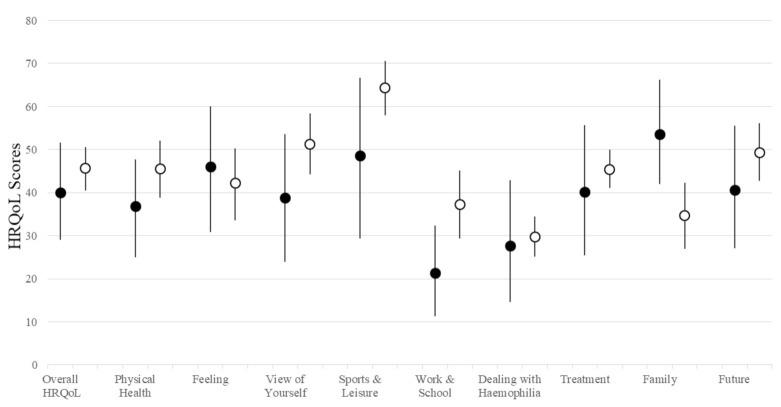
Health-related Quality of Life in Pediatric and Adult Patients with Hemophilia. HRQoL: health-related quality of life. A higher score is indicative of worse health-related quality of life (range: 0 to 100). Error bars represent 95% CI. Black: HRQoL scores of pediatric patients (*n* = 14). White: HRQoL scores of adult patients (*n* = 42). Descriptive statistics of the HRQoL scores are presented in Supplement Table S1.

Figure 1 summarizes the HRQoL scores for the adult and pediatric patients. Compared with the pediatric patients, the adult patients generally reported more problems in multiple aspects of HRQoL, especially in the domains of physical health, self-perception and work/school functioning (Figure 1). The HRQoL scores for each domain are presented descriptively in Supplement Table S1.

Table 2 summarizes the HRQoL scores in patients stratified by clinically relevant variables. Older age was associated with worse HRQoL (*r* = 0.34, *p* = 0.011), particularly in the domains of physical health (*r* = 0.37, *p* = 0.005), self-perception (*r* = 0.35, *p* = 0.008), work/school functioning (*r* = 0.59, *p* < 0.0001) and treatment satisfaction (*r* = 0.28, *p* = 0.041). Patients who received prophylactic treatment reported fewer emotional problems (38.3 [23.5] versus 56.8 [26.6]; *p* = 0.043) and fewer work/school problems (25.8 [18.9] versus 51.5 [26.3]; *p* = 0.001) than those who received on-demand therapy. Patients who resided in public housing (a marker of lower socioeconomic status) reported more impairment in family functioning (45.9 [20.5] versus 33.8 [28.3]; *p* = 0.47) and had a worse perception of the future (53.1 [23.5] versus 42.0 [21.9]; *p* = 0.041) than those who resided in private housing. Compared with adult patients who completed post-secondary education, those who attained lower than secondary school education reported worse emotional problems (34.4 [24.1] versus 55.1 [24.8]; *p* = 0.016), worse self-perception (45.4 [20.0] versus 60.9 [23.1]; *p* = 0.036) and worse work/school functioning (32.0 [24.7] versus 45.7 [22.8]; *p* = 0.045). We did not analyze the educational attainment of parents of pediatric patients because few parents had completed post-secondary education (*n* = 4).

### 3.2. Adherence

The overall non-adherence score was low in patients who received prophylactic treatment (mean [SD], 57.3 [15.8]; Supplement Table S2), suggesting satisfactory adherence to treatment. The mean scores for the adherence subscales (“Time,” “Dose,” “Plan,” “Remember”, and “Skip”) ranged from 5.5 to 8.9, which were lower than the published cutoff scores for non-adherence [30]. Higher non-adherence scores were reported for the “Time” (9.5 [4.2]) and “Communicate” (13.2 [4.4]) subscales.

A moderately strong correlation was observed between older age and non-adherence scores (overall, *r* = 0.66, *p* < 0.0001), especially for the subscales of “Remember” (*r* = 0.52, *p* = 0.001), “Skip” (*r* = 0.63, *p* < 0.0001) and “Communicate” (*r* = 0.40, *p* = 0.014) (Table 3). No significant association was identified between adherence and disease severity (in the overall cohort) or educational attainment (in adult patients).

### 3.3. Correlation between Adherence and HRQoL

Among patients who received prophylactic treatment, those who reported skipping their prophylactic treatment tended to have worse self-perception (*r* = 0.32, *p* = 0.044) and worse functioning in sports and leisure (*r* = 0.31, *p* = 0.033) (Table 4). Patients who missed prophylactic treatment also reported more problems in dealing with hemophilia (*r* = 0.31, *p* = 0.032) and lower treatment satisfaction (*r* = 0.28, *p* = 0.049).

### 3.4. Exploratory Analyses

The multivariable model (Table 5) shows that older age was associated with worse overall HRQoL (unstandardized coefficient [*B*] = 0.45, 95% confidence interval [CI] = 0.07–0.83). Adherence score, disease severity and housing type were not significantly associated with the HRQoL scores. Among adult patients, older age (*B* = 0.42, 95% CI = 0.093–0.75) and living in public housing (*B* = 10.24, 95% CI = 0.70–19.77) were associated with worse HRQoL.

The internal consistency of the Haem-A-QoL, Haemo-QoL-SF (children) and VERITAS-Pro subscales is presented in Supplement Table S3. The Cronbach’s α of the Haem-A-QoL and Haem-QoL-SF (children) ranged from 0.72 to 0.96, indicating good internal consistency. In the VERITAS-Pro, the lowest internal consistency was found in the “Dose” subscale (Cronbach’s α = 0.41). Ten potentially problematic items that required cautious interpretation were identified across these three instruments (Haem-A-QoL, *n* = 4; Haemo-QOL-SF, *n* = 2; and VERITAS-Pro, *n* = 4) (Supplement Table S3).

## 4. Discussion

We found that Chinese patients with hemophilia in Hong Kong generally reported good work/school functioning and were confident in dealing with their condition. However, they had poor perception of their physical health, functioning in sports and leisure, and self-perception. Consistent with the literature [13,34], our findings show that patients who received prophylactic treatment reported better HRQoL than those who received on-demand therapy. Among adult patients, those with lower educational attainment demonstrated worse perceived functioning. Most importantly, non-adherence to treatment was found to be negatively associated with specific aspects of HRQoL such as self-perception, sports and leisure functioning and treatment satisfaction. This finding may have implications for developing patient-centered rehabilitation interventions to improve patients’ self-efficacy in managing their lifelong health condition.

We found that the mean HRQoL score (obtained using the validated Haem-A-QoL tool) in our adult cohort (45.7 points) was comparable to that reported in a multinational study [35] involving 12 middle-income and high-income countries (47.3 points) and that reported in a Turkish study [36] (47.4 points). Notably, a wide range of Haem-A-QoL scores were reported by studies that were conducted in different countries and on patients receiving different treatments and with different disease severity [13,26,28,29,34,35,36,37,38]. For example, the mean HRQoL impairment score (45.7 points) in our study was lower than the reported estimate of 62.7 in a recent large-scale study involving 875 patients with hemophilia in China [29]. Although both studies were conducted on Chinese patients, HRQoL perception may be influenced by cultural and geographical differences, and disparities in healthcare settings, across China [29,39,40]. Although such comparisons should be interpreted cautiously owing to the small size of our study cohort, the differences in reported HRQoL may suggest there are disparities in hemophilia-related interventions, support systems and health policies across countries/regions.

Our results show that our patients perceived the worst functioning in the HRQoL domain of “Sports and Leisure.” The finding is consistent with those of other published studies that show the highest degree of HRQoL impairment in the “Sports and Leisure” domain of the Haem-A-QoL and Haem-QoL-SF tools [6,7,28,35,36,38]. Many studies reported that patients with hemophilia are less physically active than healthy controls, for reasons such as a fear of bleeding, an insufficient recognition of the benefits of exercise, and a lack of confidence in risk assessment and management [41,42]. It is also well documented that the promotion of physical activity is especially important for patients with hemophilia, to reduce their risk of fall injuries and lifestyle-related diseases [14,43,44]. This finding provides justification for adopting innovative strategies to encourage exercise in these populations, such as adventure-based and online home-based physical exercise programs [44,45], to promote physical activity in patients with hemophilia.

Our findings further show that compared with younger patients, older patients had a poorer perception of multiple aspects of their HRQoL, including perception of physical health and feelings, self-perception, and perception of the future. Multivariable analysis in adult patients also shows that older age was associated with poorer overall HRQoL. Although our study sample included patients across a wide age range, few (14%) were older than 35 years. Studies showed that the risks of developing hemophilia-related complications, including intracranial hemorrhage, joint disease and inhibitor development, increase with age [2,37,46]. We speculate that as patients age, their late complications and increasing symptom burden contribute to their declining health status and restrict their role functioning. Similar to mainland China where hemophilia treatment is in the scope of basic medical insurance reimbursement [47], the cost of the coagulation factor concentrate drugs are fully covered by the public healthcare system for hemophilia patients in Hong Kong. However, future research should still evaluate the occupational outcomes and economic burden in this population. The findings of these studies can be used to guide the development of targeted rehabilitation programs to empower patients with the skills to meet the challenges of the workforce and improve their career advancement opportunities.

The patients in our study were generally adherent to their prophylactic treatment; the scores for the VERITAS-Pro subscales, except for the “Communicate” subscale, were lower than the cutoff scores for non-adherence [30]. This suggests the need to encourage patients to more proactively communicate with their healthcare providers if they have health-related concerns. Consistent with other reports [6,36], we found that older patients may be at risk of experiencing poor functional outcomes due to non-adherence. This is because pediatric patients probably have caregivers to assist them with managing their hemophilia, while the self-infusion schedules of adult patients might be affected by work or other pressing commitments [37,48]. This suggests the need to reinforce age-appropriate education in self-management during early adolescence and throughout different stages of life with tailored supportive care and transition plans.

Poor treatment adherence was correlated with impairment in specific aspects of HRQoL. Although the overall association between adherence and HRQoL was not significant in the multivariable analysis, the benefits of treatment adherence in improving health and psychosocial outcomes in patients with hemophilia are well documented in the literature [15,17,18,31,48]. In addition to improving functional outcomes, one Chinese study also showed that the suboptimal use of coagulation factor concentrates was associated with higher direct medical cost and healthcare use [49]. The findings collectively underscore the importance of developing strategies to improve adherence and optimize usage of prophylactic treatment in this patient cohort. For example, the development of new clotting factor drugs with a longer half-life may reduce infusion-schedule burdens and increase patient acceptance of prophylaxis [2,50], although the cost and accessibility of such novel treatments may be a challenge. Various technologies can also be used as platforms to promote health education and protective health behaviors [51,52,53]. Our future work includes developing a telehealth intervention [53] to improve treatment adherence and help clinicians formulate or modify treatment plans in a timely manner for patients with hemophilia in Hong Kong.

Our results should be interpreted in the light of several limitations. First, the sample size was small, which was expected because hemophilia is a rare disease. However, we recruited patients through an NGO, and the response rate was high. This approach helped to establish the sampling frame and likely reduced the risk of selection bias. Our sample is also heterogeneous in terms of age, disease severity and treatment strategies; therefore, the overall adherence and HRQoL findings should be interpreted with caution. As this study was conducted through an NGO, we did not have access rights to detailed medical information on the patients’ treatment and comorbidities. Future work includes targeting a more representative sample with a larger cohort and the collection of comprehensive HRQoL and functional performance data so that clinically relevant factors (e.g., types of clotting factors, dose intensity, short vs long half-lives of clotting factors, and the presence of inhibitors) on health outcomes can be analyzed. Fortunately, the use of prophylactic treatment in patients with hemophilia has decreased the demand for blood transfusion. Consequently, the risk of blood transfusion-related infections (e.g., Hepatitis C virus infection) has reduced drastically due to the replacement of recombinant products with plasma-derived products and the successful implementation of blood management programs worldwide [54]. It is worthwhile to evaluate in-depth the health outcomes among the minority of patients on antiviral therapy.

Lastly, the internal validity of the findings should be interpreted cautiously, as the participants’ self-reports of treatment adherence might have been influenced by social desirability bias and differences between patients’/caregivers’ and clinicians’ concepts of adherence. We also did not evaluate other factors that might influence HRQoL or treatment adherence (e.g., patients’ financial status, personal values, and religion/spirituality). As there is currently no gold standard for measuring adherence to prophylactic treatment in patients with hemophilia, we suggest that future work uses a combination of subjective methods (interviews and treatment diaries) and objective methods (reviews of dispensing records and measurements of blood concentrations of clotting factors) to collect adherence data with the best possible accuracy [55]. Although our exploratory analysis showed satisfactory reliability and item-scale correlation, the psychometric properties of the Haem-A-QoL, Haemo-QOL-SF and VERITAS-Pro might not sufficiently measure the variables of interest in our Chinese population. Future study should also consider using other validated tools, such as the widely used HRQoL tool EQ-5D [5,10], to facilitate comparison of outcomes across multinational studies. Despite these limitations, this study has achieved its aim of generating preliminary evidence on clinical factors associated with poor treatment adherence and their impact on HRQoL in Chinese patients with hemophilia in Hong Kong. This fills a key gap in the literature on the current state of available supportive care for this patient cohort.

## 5. Conclusions

Our results show that patients who were older, had lower education attainment, and received on-demand treatment had poorer perception of physical health, sports and leisure, and treatment satisfaction. Encouragingly, the patients who received prophylactic infusions demonstrated satisfactory treatment adherence. Our findings also suggest that improving adherence may lead to better HRQoL, especially in older patients. It is anticipated that addressing modifiable behavioral factors may contribute to better self-care, enjoyment, and participation in society among patients with hemophilia. Our future work will also include evaluating the occupational needs and limitations prospectively in this population.

## Figures and Tables

**Table 1:** Demographics and Clinical Characteristics of Study Cohort.

	Total (*n* = 56)	Adult Patients (*n* = 42)	Pediatric Patients (*n* = 14)	Parent of Pediatric Patients (*n* = 14)
Age (years) mean (SD) [range]	30.4 (17.4) [5.2–68.4]	37.2 (14.5) [17.5–68.4]	10.0 (2.8) [5.2–15.1]	41.5 (6.6) [34.7–55.9]
	*n* (%)	*n* (%)	*n* (%)	*n* (%)
Sex				
Male	56 (100.0)	42 (100.0)	14 (100.0)	2 (14.3)
Female	0 (0)	0 (0)	0 (0)	12 (85.7)
Diagnosis				
Hemophilia A	42 (75.0)	32 (76.2)	10 (71.5)	/
Hemophilia B	10 (17.9)	7 (16.7)	3 (21.4)	/
Missing/unsure	4 (7.1)	3 (7.1)	1 (7.1)	/
Treatment type				
Prophylaxis + on-demand therapy	38 (67.8)	26 (61.9)	12 (85.7)	/
On-demand therapy	17 (30.4)	15 (35.7)	2 (14.3)	/
Missing/unsure	1 (1.8)	1 (2.4)	0 (0)	/
Disease Severity				
Mild	4 (7.1)	4 (9.5)	0 (0)	
Moderate	16 (28.6)	14 (33.4)	2 (14.3)	/
Severe	33 (58.9)	22 (52.3)	11 (75.6)	/
Missing/ unsure	3 (5.4)	2 (4.8)	1 (7.1)	/
Highest Education Attainment				
Secondary school or below	30 (53.6)	16 (38.1)	14 (100)	10 (71.4)
Post-secondary school or above	26 (46.4)	26 (61.9)	0 (0)	4 (28.6)
Types of Housing				
Public housing	26 (46.4)	21 (50.0)	5 (35.7)	/
Private housing	30 (53.6)	21 (50.0)	9 (64.3)	/

SD: standard deviation.

**Table 2:** Summary and Univariate Comparison of Health-related Quality of Life Outcomes across Clinically Relevant Subgroups.

	Total *	Subscales *								
	Total	Physical Health	Feeling	View of Yourself	Sports and Leisure	Work and School	Dealing with Hemophilia	Treatment	Family	Future
Age #	***p* = 0.011**	***p* = 0.005**	*p* = 0.17	***p* = 0.008**	*p* = 0.058	***p* < 0.0001**	*p* = 0.34	***p* = 0.041**	***p* = 0.025**	*p* = 0.054
	*r* = 0.34	*r* = 0.37	*r* = 0.19	*r* = 0.35	*r* = 0.26	*r* = 0.59	*r* = 0.13	*r* = 0.28	*r* = −0.30	*r* = 0.26
Disease severity	*p* = 0.71	*p* = 0.21	*p* = 0.52	*p* = 0.69	*p* = 0.85	*p* = 0.94	*p* = 0.54	*p* = 0.33	*p* = 0.37	*p* = 0.48
Mild-moderate	41.8 (15.3)	37.8 (22.3)	38.2 (22.7)	45.8 (19.6)	60.3 (23.1)	32.2 (23.2)	29.9 (17.4)	43.4 (17.2)	35.3 (25.6)	44.9 (19.7)
Severe	45.1 (18.6)	45.6 (21.1)	44.4 (27.3)	48.6 (25.9)	61.2 (24.2)	32.4 (23.1)	28.7 (20.1)	44.4 (20.0)	41.5 (26.6)	48.5 (24.3)
Treatment type	***p* = 0.039**	*p* = 0.24	***p* = 0.043**	*p* = 0.34	*p* = 0.31	***p* = 0.001**	*p* = 0.53	*p* = 0.17	*p* = 0.96	*p* = 0.21
Prophylaxis	41.4 (16.8)	42.4 (19.5)	38.3 (23.5)	45.0 (23.0)	58.1 (24.9)	25.8 (18.9)	28.6 (19.8)	42.4 (19.1)	39.5 (25.8)	44.2 (20.1)
On-demand	52.1 (18.6)	48.2 (25.9)	56.8 (26.6)	55.8 (25.6)	65.3 (24.6)	51.5 (26.3)	30.4 (18.6)	49.4 (18.5)	40.1 (26.2)	55.1 (28.1)
Housing type	*p* = 0.073	*p* = 0.13	*p* = 0.18	*p* = 0.25	*p* = 0.51	*p* = 0.11	*p* = 0.46	*p* = 0.71	***p* = 0.047**	***p* = 0.041**
Public	48.8 (17.5)	48.2 (24.1)	48.5 (28.6)	53.1 (24.5)	63.4 (23.3)	38.2 (25.4)	30.1 (16.7)	43.7 (19.5)	45.9 (20.5)	53.1 (23.5)
Private	40.4 (17.8)	39.3 (19.9)	38.6 (23.3)	44.0 (23.0)	58.0 (25.8)	29.0 (23.1)	28.4 (21.2)	44.3 (19.5)	33.8 (28.3)	42.0 (21.9)
Education Ɨ (Adult patient)	***p* = 0.036**	*p* = 0.066	***p* = 0.016**	***p* = 0.036**	*p* = 0.51	***p* = 0.045**	*p* = 0.94	*p* = 0.89	*p* = 0.35	*p* = 0.16
<Secondary	52.8 (16.2)	53.1 (23.9)	55.1 (24.8)	60.9 (23.1)	67.2 (17.7)	45.7 (22.8)	29.2 (17.5)	46.5 (16.0)	38.7 (22.8)	57.2 (23.5)
Post-secondary	41.4 (15.7)	41.0 (20.0)	34.4 (24.1)	45.4 (20.0)	62.7 (20.6)	32.0 (24.7)	30.6 (15.4)	43.8 (13.7)	32.2 (26.1)	44.4 (18.8)

* Health-related quality of life total score and subscale scores are presented as mean (standard deviation). A higher score is indicative of worse health-related quality of life (range: 0 to 100). *#* Age was analyzed as a continuous variable. Strength of correlation is presented as correlation coefficient (*r*). Ɨ Analysis on highest education attainment was conducted among adult survivors only. Analysis on parental education was not conducted in pediatric patients due to the small sample size (*n* = 14). Bold denotes statistically significant values (*p*  <  0.05).

**Table 3:** Summary and Univariate Comparison of Treatment Adherence across Clinically Relevant Subgroups among Patients who Received Prophylactic Treatment.

	Total *	Subscales *					
	Range: 24–120	Time Range: 4–20	Dose Range: 4–20	Plan Range: 4–20	Remember Range: 4–20	Skip Range: 4–20	Communicate Range: 4–20
Age #	***p* < 0.0001**	***p* = 0.001**	*p* = 0.086	*p* = 0.61	***p* = 0.001**	***p* < 0.0001**	***p* = 0.014**
	*r* = 0.66	*r* = 0.51	*r* = 0.28	r= −0.09	*r* = 0.52	*r* = 0.63	*r* = 0.40
Disease severity	*p* = 0.46	*p* = 0.21	*p* = 0.68	*p* = 0.10	*p* = 0.66	*p* = 0.46	*p* = 0.10
Mild-moderate	59.6 (11.9)	11.0 (4.2)	8.8 (4.0)	10.0 (2.7)	9.0 (4.4)	9.2 (4.2)	11.6 (3.4)
Severe	56.1 (17.3)	9.0 (4.2)	8.7 (2.7)	8.2 (3.4)	8.3 (4.2)	8.2 (4.4)	13.7 (4.8)
Housing type	*p* = 0.38	*p* = 0.38	***p* = 0.041**	*p* = 0.38	*p* = 0.76	*p* = 0.34	*p* = 0.39
Public	60.0 (16.9)	8.9 (3.8)	9.6 (2.9)	9.4 (3.3)	8.9 (4.5)	9.3 (4.6)	13.9 (4.1)
Private	67.2 (17.4)	12.4 (5.0)	9.8 (4.7)	10.2 (1.9)	11.2 (5.7)	10.4 (5.3)	13.2 (4.0)
Education Ɨ (Adult patients)	*p* = 0.85	*p* = 0.40	*p* = 0.89	*p* = 0.85	*p* = 0.98	*p* = 0.94	*p* = 0.68
<Secondary	62.6 (12.7)	9.5 (3.3)	9.1 (3.3)	8.9 (2.9)	9.8 (3.5)	10.0 (4.3)	15.4 (2.8)
Post-secondary	64.2 (14.5)	11.6 (4.1)	9.3 (3.6)	8.7 (3.6)	10.2 (4.4)	10.0 (4.4)	14.6 (3.6)

* Adherence total score and subscale scores are presented as mean (standard deviation). A higher score is indicative of worse adherence. *#* Age was analyzed as a continuous variable. Strength of correlation is presented as correlation coefficient (*r*). Ɨ Analysis on patients’ highest education attainment was conducted among adult survivors only. Analysis on parental education was not conducted in pediatric patients due to the small sample size (*n* = 14). Note: Bold denotes statistically significant values (*p*  <  0.05).

**Table 4:** Correlation between Treatment Adherence and Health-related Quality of Life Outcomes.

HRQoL #	Treatment Adherence *						
	Total Adherence Score	Time	Dose	Plan	Remember	Skip	Communicate
Total HRQoL score	−0.04	−0.25	0.05	0.17	0.06	0.16	−0.26
Physical Health	−0.11	−0.059	0.01	−0.08	0.10	0.29	0.17
Feeling	−0.06	0.32 Ɨ	0.12	0.07	0.26	0.15	−0.14
View of Yourself	0.16	0.23	0.21	0.04	0.19	0.32 Ɨ	−0.06
Sport and Leisure	−0.03	0.07	0.24	0.07	0.02	0.31 Ɨ	0.20
Work and School	0.92	0.26	0.15	0.20	−0.002	0.09	0.007
Dealing with Hemophilia	0.17	−0.08	−0.03	0.31 Ɨ	0.10	0.16	−0.13
Treatment	0.21	−0.24	−0.003	0.28 Ɨ	0.08	0.11	−0.18
Future	0.07	0.15	0.14	−0.016	0.26	0.08	0.10
Family	−0.18	−0.001	0.33 Ɨ	0.26	0.09	0.11	−0.19

HRQoL: Health-related quality of life. * A higher score is indicative of worse adherence. # A higher score is indicative of worse health-related quality of life. Ɨ denotes statistically significant values (*p*  <  0.05). Strength of correlation is presented as correlation coefficient (*r*).

**Table 5:** Exploratory Multivariable Analysis of Factors Associated with Health-related Quality of Life.

	HRQoL Scores * (All Patients)			HRQoL Scores * (Adult Patients Only)		
	*B*	95% CI	*p*	*B*	95% CI	*p*
Adherence score #	0.15	(−0.19, 0.50)	0.38	0.21	(−0.13, 0.54)	0.22
Age #	0.45	(0.07, 0.83)	**0.021**	0.42	(0.093, 0.75)	**0.014**
Disease severity						
Mild-moderate				*ref*		
Severe	0.31	(−0.69, 1.31)	0.54	0.90	(−8.49, 10.29)	0.85
Housing type						
Private				*ref*		
Public	8.30	(−1.39, 17.97)	0.091	10.24	(0.70, 19.77)	**0.036**
Education Ɨ (Adult patients only)						
<Secondary	/	/	/	*ref*		
Post-secondary	/	/	/	−1.22	(−10.8, 8.35)	0.80

*B*: unstandardized coefficient; HRQoL: health-related quality of life; 95% CI: 95% confidence interval. * The dependent variable refers to the overall health-related quality of life score (continuous variable). A higher score is indicative of worse health-related quality of life. Separate models were run separately within the overall cohort and adult patients. Multivariable analysis was not conducted on pediatric patients due to the small sample size (*n* = 14). *#* Age and overall adherence score was analyzed as a continuous variable. A higher score is indicative of worse adherence. Ɨ Analysis on patients’ highest education attainment was conducted among adult survivors only. Bold denotes statistically significant values (*p*  <  0.05).

## Data Availability

The datasets generated during and/or analyzed during the current study are not publicly available due to identifiable data and privacy/confidentiality concerns but are available from the corresponding author on reasonable request.

## Supplementary Materials

The following supporting information can be downloaded at: https://www.mdpi.com/article/10.3390/ijerph19116496/s1. Table S1: Health-Related Quality of Life Scores; Table S2: Adherence Scores among Patients Who Received Prophylactic Treatment; Table S3: Internal Consistency among Subscales.
