# Supplementary material for: Treatment Adherence and Health-Related Quality of Life in Patients with Hemophilia in Hong Kong
Source: Int J Environ Res Public Health. 2022 May 26;19(11):6496. doi: 10.3390/ijerph19116496 (PMC9180237; doi:10.3390/ijerph19116496)
Supplement: Supplementary file 1 [file ijerph-19-06496-s001.zip › ijerph-1679578-supplementary.pdf]

Supplement Table S1: Health-related Quality of Life Scores

	Total (N = 56)		Pediatric patients (n = 14)		Adults patients (n = 42)	
**Scores***	Mean	SD	Mean	SD	Mean	SD
**Overall HRQoL**	44.3	18.0	40.0	21.5	45.7	16.7
Physical Health	43.4	22.2	36.9	22.1	45.6	22.1
Feeling	43.2	26.1	46.1	26.9	42.3	26.1
View of Yourself	48.2	23.9	38.8	26.9	51.3	22.3
Sport and Leisure	60.5	24.6	48.7	34.2	64.4	19.4
Work and School	33.3	24.4	21.4	20.0	37.2	24.7
Dealing with Haemophilia	29.2	19.1	27.7	26.3	29.8	16.5
Treatment	44.0	19.3	40.2	19.2	45.3	15.0
Family	39.4	25.5	53.6	22.8	34.7	24.8
Future	47.1	23.2	40.7	27.8	49.3	21.4

HRQoL: health-related quality of life; SD: standard deviation
*A higher score is indicative of worse health-related quality of life (range: 0 to 100).

Supplement Table S2: Adherence Scores Among Patients Who Received Prophylactic Treatment

	Total (N = 38)		Pediatric patients (n = 12)		Adults patients (n = 26)	
**Scores [Cutoff score for "non-adherence"]***	Mean	SD	Mean	SD	Mean	SD
**Overall adherence [57]**	57.3	15.8	43.3	10.2	63.7	13.8
Time [11]	9.5	4.2	6.4	3.1	10.9	3.9
Dose [7]	8.9	3.1	8.1	1.8	9.2	3.5
Plan [9]	8.7	3.2	8.7	3.1	8.7	3.3
Remember [11]	8.6	4.2	5.3	1.9	10.0	4.1
Skip [11]	8.5	4.2	5.2	1.3	10.0	4.3
Communicate [10]	13.2	4.4	9.6	4.5	14.8	3.3

SD: standard deviation

* A higher score is indicative of worse adherence. Score range for overall adherence: 24 – 120 Score range for all subscales: 4 – 20

Cutoff scores in square brackets refer to published thresholds defining "non-adherence" by the developers of the Validated Hemophilia Regimen Treatment Adherence Scale-Prophylaxis (VERITAS-Pro)

Supplement Table S3: Internal Consistency among Subscales

	Cronbach's α	Corrected Item-scale Correlation	Potentially Problematic Items *
**Haem-A-QoL**			
Physical Health	0.88	0.53 – 0.85	*“I need more time to get ready”*
Feeling	0.89	0.65 – 0.83	*“I felt excluded”*
View of Yourself	0.88	0.70 – 0.77	
Sports and Leisure	0.72	0.46 – 0.77	*“I didn’t have the freedom to travel where I wanted”*
Work and School	0.87	0.67 – 0.82	
Dealing with Haemophilia	0.65	0.47 – 0.67	*“I was dependent on the factor concentrate because of my hemophilia”*
Future	0.74	0.62 – 0.73	
Family	0.82	0.63 – 0.66	
**Haemo-QoL-SF**			
Physical Health	0.91	0.79 – 0.89	
Feeling	0.93	0.73 – 0.89	
View of Yourself	0.90	0.68 – 0.84	
Sports and Leisure	0.96	0.86 – 0.93	
Work and School	0.91	0.59 – 0.79	*“I was able to participate in school, even with my hemophilia”*
Dealing with Haemophilia	0.96	0.73 – 0.91	
Treatment	0.87	0.72 – 0.82	
Future	0.93	0.75 – 0.93	
Family	0.72	0.52 – 0.67	*“My parents didn’t allow me to do certain things because of my hemophilia”*
**VERITAS-Pro**			
Time	0.94	0.76 – 0.88	
Dose	0.41	0.34 – 0.52	*“I infuse at a lower dose than prescribed”*<br>*“I increase or decrease the dose without calling the hospital”*
Plan	0.69	0.53 – 0.71	*“I ran out of factors and supplies before I order more”*
Remember	0.82	0.64 – 0.75	

Skip	0.95	0.85 – 0.91	
Communicate	0.76	0.53 – 0.86	*“I make treatment decisions myself rather than calling the hospital”*

Haem-A-QoL: Haemophilia Quality of Life Questionnaire for Adults; Haemo-QOL-SF: Haemophilia Quality of Life Questionnaire for Children Short Form; VERITAS-Pro: Validated Hemophilia Regimen Treatment Adherence Scale-Prophylaxis

* Refers to items with a weak corrected item-scale correlation < 0.6
